# Supplementary material for: Growth Differentiation Factor 15 as a Predictor of the No-Reflow Phenomenon in Patients with ST-Segment Elevation Myocardial Infarction
Source: J Clin Med. 2022 Dec 29;12(1):245. doi: 10.3390/jcm12010245 (PMC9821761; doi:10.3390/jcm12010245)
Supplement: Supplementary file 1 [file jcm-12-00245-s001.zip › jcm-2079778-supplementary.pdf]

Table S1. Pharmacological treatment at discharge.

	**All patients N=80**	**No-reflow group N=19**	**Reflow group N=61**	p
Clopidogrel	92%	88%	93%	0.189
Prasugrel	5%	6%	5%	0.915
Ticagrelor	3%	6%	2%	0.359
Acetylsalicylic acid	100%	100%	100%	
ACE-inhibitors	94%	89%	95%	0.349
Beta-blockers	95%	94%	95%	0.915
Statins	96%	94%	97%	0.662
Mineralocortisteroid Antagonists	48%	61%	44%	0.214
Diuretics	47%	67%	41%	0.056

ACE - angiotensin converting enzyme.

Table S2. Clinical characteristics.

	All patients N=80	No-reflow group N=19	Reflow group N=61	p
Male gender	73%	68%	74%	0.653
Age (years)	64.75 (11.72)	71.95 (10.50)	62.51 (11.24)	0.002
Time pain to door (minutes)	200 (300)	172 (450)	100 (183.5)	0.001
Anterior infarction	52%	63%	49%	0.292
Cardiac arrest before/during hospitalization	7.5%	11%	7%	0.572
Ejection fraction of left ventricle (%)	39.30 (8.95)	37.11 8.94)	39.98 (8.91)	0.223
Atrial fibrillation during hospitalization	8.75%	21%	5%	0.030
BMI	27.65 (4.39)	28.56 (4.53)	27.38 (4.36)	0.320
e-GFR (ml/minute)	90.12 (32.59)	76.87 (32.21)	94.04 (31.91)	0.049
Systolic blood pressure on admission (mmHg)	136.94 (25.39)	123.84 (22.68)	141.02 (24.96)	0.009
Diastolic blood pressure on admission (mmHg)	83.21 (14.91)	76.89 (12.13)	85.18 (15.22)	0.033
Heart rate at admission (beats/minute)	78.49 (18.99)	81.26 (25.43)	77.62 16.63)	0.469

BMI - body mass index, e-GFR - estimated glomerular filtration rate.
